# Supplementary material for: Complexome profiling of the Chlamydomonas psb28 mutant reveals TEF5 as an early PSII assembly factor
Source: Plant Cell. 2025 Mar 17;37(6):koaf055. doi: 10.1093/plcell/koaf055 (PMC12164590; doi:10.1093/plcell/koaf055)
Supplement: koaf055_Supplementary_Data [file koaf055_supplementary_data.zip › psb28_interactive.zip!/plots/intensity/qualityPlots/peptides/psbJ_Cre-1.g2716960.t1.p1.html]

### Cre-1.g2716960.t1.p1

Trivial name: psbJ  
SeqCoverage [median]: 18.00  
Mass: 5.72 kDa  
  
 

| Sequence | MissedCleavage | Mass [Da] | wtIntensity | muIntensity | Score |
| --- | --- | --- | --- | --- | --- |
| MSNTGTTGR | 0 | 923.41 | 264.38 | 0.00 | 66.62 |
| SNTGTTGR | 0 | 792.37 | 278083.97 | 117842.04 | 147.36 |
|  |  |  | 278348.4 | 117842.0 |  |
